# Posttraumatic Symptoms as Predictors of Engagement With a Mobile App for Coping After Military Sexual Trauma: Public Usage Data Analysis Study

**DOI:** 10.2196/85098

**Published:** 2026-05-07

**Authors:** Shilpa Hampole, Shannon McCaslin-Rodrigo, Valerie Carr, David Schuster, Sofia Reyes, Sarah Senti, Kelly M Ramsey, Jason E Owen, W Christopher Skidmore, Amy E Street, Katherine Taylor, Margaret-Anne Mackintosh

**Affiliations:** 1 Dissemination and Training Division National Center for PTSD VA Palo Alto Health Care System Menlo Park, CA United States; 2 Department of Psychiatry and Behavioral Sciences School of Medicine Stanford University Stanford, CA United States; 3 Department of Psychology San José State University San Jose, CA United States; 4 Office of Mental Health Veterans Health Administration Washington, DC United States; 5 VA Boston Healthcare System Boston, MA United States; 6 Department of Psychiatry Chobanian & Avedisian School of Medicine Boston University Boston, MA United States

**Keywords:** military sexual trauma, posttraumatic stress disorder, trauma, veterans, mental health, mobile app, mHealth, digital health, public health

## Abstract

**Background:**

Military sexual trauma (MST) can have significant adverse effects on mental health and well-being, often leading to posttraumatic stress disorder (PTSD) symptoms and maladaptive beliefs. Although effective psychotherapies exist, stigma, confidentiality concerns, and systemic barriers often hinder help-seeking among service members and veterans. Mobile mental health apps offer an accessible and anonymous support alternative, potentially addressing such barriers. However, app effectiveness depends on user engagement and emerging evidence suggests that engagement may be shaped by symptom severity.

**Objective:**

This retrospective observational study aimed to explore the relationship between posttraumatic symptom severity and user engagement with Beyond MST (US Department of Veterans Affairs [VA] National Center for PTSD), an app for individuals who experienced MST. Specific aims included (1) characterizing trauma-related symptom levels and app engagement among users who completed in-app assessments, and (2) evaluating how PTSD symptom severity, negative posttraumatic cognitions, and mental well-being relate to objective measures of engagement.

**Methods:**

Anonymous usage data from 27,517 users collected between March 11, 2021 and July 29, 2024, were analyzed. Three subsamples were identified: those who completed the in-app PTSD checklist for *DSM-5* (*Diagnostic and Statistical Manual of Mental Disorders* [*Fifth Edition*]; PCL-5, n=3689), the Posttraumatic Maladaptive Beliefs Scale (PMBS; n=2197), and the Warwick-Edinburgh Mental Well-Being Scale (WEMWBS; n=2160). Engagement metrics included duration of use (ie, days of use and minutes of use), frequency of feature access (ie, coping tool and psychoeducation access), and frequency of feature use (ie, total assessment completions). Regression analyses, including quadratic terms, were conducted to evaluate how symptom severity and well-being levels influenced engagement and identify possible curvilinear trends.

**Results:**

Median engagement levels ranged across subsamples as follows: 3-4 days of use (IQR 5-6), 22-30 minutes of use (IQR 33.7-42.9), 1-5 feature accesses (IQR 6-9), and 2-3 assessment completions (IQR 2). Subsamples were highly symptomatic. Analyses revealed that moderate PTSD symptom and negative posttraumatic cognition severity were associated with higher engagement relative to users with very low and very high symptom levels, particularly for days of use and frequency of coping tool access. Conversely, higher mental well-being scores were generally linked to increased app engagement with linear effects. Effect sizes were small, suggesting limited clinical impact.

**Conclusions:**

This study highlights the possible challenges in engaging highly symptomatic individuals with digital mental health interventions. Although Beyond MST successfully reaches its targeted population, very low or high symptom levels and lower well-being may hinder sustained engagement. These findings suggest that symptom levels should be considered in app development (ie, personalization) and when integrating apps into professional care. Interpretation is limited by the anonymous nature of the data, which prevented characterization of users and their trauma histories. Further research is needed to clarify how symptom patterns influence engagement, especially in trauma contexts.

**International Registered Report Identifier (IRRID):**

RR2-10.31979/etd.882a-5fcx

## Introduction

### Prevalence of Military Sexual Trauma

Experiences of sexual assault or harassment during military service, known as military sexual trauma (MST) [1], are common and can have lasting negative effects on military service members and veterans, including impacts on military preparedness, postservice readjustment, and long-term mental and physical health. In a national survey of 20,563 recent veterans conducted from 2009 to 2011 by the US Department of Veterans Affairs (VA), approximately 41% of women and 4% of men reported experiences of MST [2]. However, the actual prevalence of MST may be even higher (eg, as high as 74% in women veterans [3]) due to underreporting and the substantial heterogeneity in definitions and methods for assessing MST across studies [4,5].

### Implications of MST on Mental Health

MST can have profound mental health implications, often leading to severe psychological distress, poor psychosocial functioning, and reduced quality of life. The most common mental health condition associated with MST is posttraumatic stress disorder (PTSD) [5]. Military personnel are at high risk for developing PTSD due to combat and other traumatic exposures experienced during military service, and experiencing MST further exacerbates this risk [6]. In addition, experiences of MST are associated with other mental health conditions such as substance use [7], depression, and suicidality [8], all of which compound the psychological burden on affected individuals. These findings underscore the critical need for interventions that support individuals coping with the aftereffects of MST.

MST, like other forms of trauma, can also lead to increased dysfunctional beliefs or maladaptive thinking patterns. Research suggests that negative posttraumatic cognitions (NPCs, ie, distorted or maladaptive thoughts and beliefs that develop following trauma) play a crucial role in the mechanisms of action of PTSD [9,10]. Specifically, trauma can disrupt an individual’s core beliefs about themselves, others, or the world, which can lead to maladaptive beliefs (eg, “I don’t feel safe anywhere anymore” [11]) and loss of meaning in life [9,12]. Even when controlling for other posttraumatic risk factors, NPCs can intensify other PTSD symptoms such as avoidance and hypervigilance [10]. Overall, NPCs can hinder recovery after traumatic events and may be especially problematic following experiences of MST. Notably, MST is frequently associated with feelings of self-stigma, or the internalization of negative stereotypes that can lead to guilt, shame, and self-blame. This can manifest in NPCs such as, “The event happened to me because of the sort of person I am,” or “Somebody else would not have gotten into this situation” [13]. The distinctive nature of the experience of MST highlights the importance of tailored, evidence-based approaches that address NPCs and other symptoms of sexual trauma.

### Implications of MST on Help-Seeking

Although numerous psychotherapy interventions exist to address symptoms of PTSD following traumatic experiences such as MST (eg, Cognitive Processing Therapy, Prolonged Exposure, and Acceptance and Commitment Therapy) [14], individuals who have experienced sexual trauma face significant barriers to help-seeking. Societal stigma around sexual trauma can translate into fear of judgment in survivors, and victim-blaming and disbelief from others. For example, one study found that higher self-stigma is associated with a lower likelihood of disclosing MST in health care screenings [15]. In addition, the setting in which MST occurs (ie, service members living in close quarters on a long-term basis; hierarchical rank structures) can produce fears around subsequent violence, work retaliation, and ostracism by colleagues [16]. Consequently, negative institutional experiences may prompt confidentiality concerns, feelings of distrust toward providers, and discomfort disclosing MST experiences in health care settings [17-19]. These kinds of barriers to help-seeking, nondisclosure with providers, and underuse of health care services can reduce an individual’s opportunities for recovery.

### Apps for Self-Managing Mental Health

Fortunately, widely accessible technology-based solutions, such as mental health apps, have the potential to expand access to psychoeducation, coping tools, and resources for self-management purposes [20]. They can also bolster patient autonomy, offer 24/7 support when traditional care is unavailable, and preserve anonymity to mitigate stigma—benefits that prove especially valuable for individuals coping with sensitive traumatic events.

Increasingly, researchers have demonstrated the effectiveness of mental health apps in improving coping skills, reducing mental health symptoms, and enhancing users’ sense of well-being and quality of life across populations and mental health challenges [21]. The PTSD Coach app, developed by the US Department of Veterans Affairs (VA) National Center for PTSD, is one well-researched example that has demonstrated feasibility, acceptability, and effectiveness across 16 studies [22]. Digital mental health technologies for addressing specific trauma types, such as sexual trauma and MST [23,24], have also emerged. For example, apps for sexual violence (especially intimate partner violence) are widely available, but typically serve informational, preventative, or peer support purposes [25-27].

The Beyond MST app (US Department of Veterans Affairs [VA] National Center for PTSD) is uniquely designed to help users self-manage MST-related psychosocial challenges (eg, mental health symptoms, behavioral issues, and relationship challenges), enhance their general well-being, and find hope via evidence-based psychoeducation and coping tools [28,29]. To date, no prior research on digital mental health technologies for individuals who experienced MST, including Beyond MST, has been conducted. While usability and effectiveness studies are important, research is also needed to identify factors that may limit users’ engagement with app content and consequently impact their effectiveness [30].

### App Engagement

Low levels of user engagement with mental health technologies in real-world, nonresearch settings are a significant problem, as many users discontinue use after only a few interactions. For instance, a review of 93 popular mental health apps revealed a median 15-day user retention rate of just 3.9% (IQR 10.3%) [31]. This pattern mirrors the issue of psychotherapy dropout, which can impede patient recovery; similarly, insufficient engagement may hinder users from achieving their full therapeutic benefit.

While early research on digital mental health technology engagement primarily examined its link to health outcomes, recent efforts have shifted toward uncovering the underlying factors that impede or promote user engagement. These include setting-, intervention-, and user-related predictors. For instance, a large body of research has examined intervention-level factors such as the addition of human coaching, gamification, and persuasive technology [30]. However, user-level predictors—particularly symptom severity—remain underexplored.

A systematic review of 208 studies on digital mental health interventions found that only 19 studies examined the relationship between symptom severity and objective measures of user engagement. Most indicated that higher symptom severity, especially related to depression and fatigue, was linked to lower engagement [32]. Yet, findings are mixed: another review reported that while 2 studies associated greater symptom severity with increased usage, 3 found the opposite [30]. Conflicting results may be due to the range of mental health conditions (eg, depression, anxiety, bipolar disorder, and schizophrenia) and digital interventions (eg, web-based services, telehealth programs, and mobile apps) assessed, as well as diversity in the engagement measures used.

The relationship between symptom severity and mental health app engagement may be further complicated by potential curvilinear patterns [30], yet very few studies have modeled symptom severity as a continuous predictor that includes quadratic (or higher-order) terms, leaving such nonlinear relationships largely untested. There is some evidence of possible nonlinear relationships between depression symptoms and mental health app engagement, with moderate symptom levels associated with relatively higher engagement, and mild and severe symptom levels associated with relatively lower engagement (eg, [33]). Possible inverted-U patterns may reflect varying barriers across symptom levels—for instance, low perceived need among users with mild symptoms and increased cognitive burden among users with severe symptoms.

Furthermore, research exploring whether posttraumatic symptom severity predicts engagement with digital mental health technologies remains limited. While some evidence suggests that higher PTSD symptoms are associated with greater intentions to use such technologies, self-reported intentions may not translate into actual behavior (eg, [32,34]). This underscores the importance of analyzing objective engagement metrics. For instance, usage data from COVID Coach (US Department of Veterans Affairs [VA] National Center for PTSD)—an app offering coping tools and resources during the COVID-19 pandemic—revealed that users with significant PTSD symptoms were more likely to use the app for more days on average than those with subthreshold symptoms [33]. Taken together, the relationship between symptom severity and engagement with digital health technologies is unclear and may vary based on symptom type and engagement measures used. Further research is needed to delineate the relationships between trauma-related symptom measures and engagement with trauma-focused apps, especially following experiences of MST where no research has been conducted.

### Study Objectives

This retrospective observational study is the first to evaluate a mental health app specifically designed for veterans and service members who have experienced MST. Given the limited research in this area, an exploratory approach was taken to (1) characterize trauma-related symptom levels and app engagement among subsamples of users who completed in-app assessments, and (2) for those subsamples, examine the relationship between trauma-related measures and objective app engagement measures. Usage data from a large sample of Beyond MST app users were processed and analyzed. Two primary predictors were examined: PTSD symptom severity and the severity of NPCs. Mental well-being was included as a secondary predictor to serve as a comparator. App engagement was measured via 3 types of variables: measures of duration of app use, frequencies of feature access, and frequency of feature use. Overall, findings may enhance our understanding of trauma-related predictors of digital mental health engagement among real-world users coping with MST.

## Methods

### Description of the Beyond MST Mobile App

The Beyond MST app was developed by the VA National Center for PTSD in collaboration with the national VA MST Support Team [28,29]. It is also part of a larger portfolio of mental health apps developed and maintained by the National Center for PTSD [35]. Beyond MST can be used for self-care purposes (eg, to self-manage MST-related symptoms) or in conjunction with professional health care. However, the app is not intended to replace professional therapy or treatment. While app content was curated for individuals who experienced MST, it may also be helpful to those who have experienced other types of sexual trauma outside of military settings (eg, intimate partner violence).

Beyond MST is publicly available and can be downloaded for free from the Google Play and Apple App stores on smartphones or tablets. It was first released in March 2021 and has since undergone ongoing maintenance and app updates. Once downloaded, the app can be used without an internet connection. Additionally, no user account is required. The app is also private and secure, meaning all usage data collected are anonymous, and users can opt out of providing usage data.

**Figure 1:**
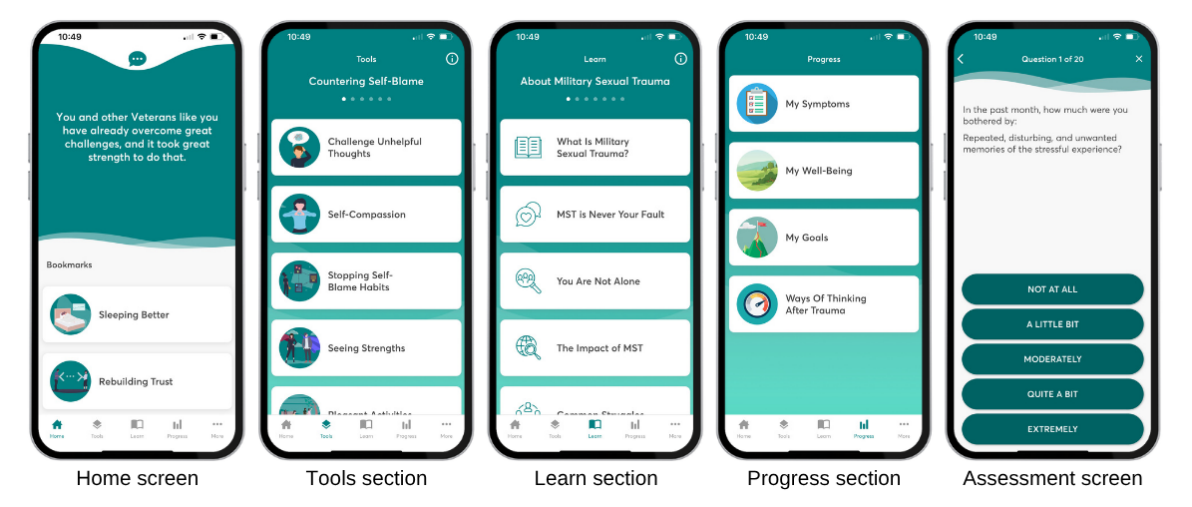
Screenshots from the Beyond MST (military sexual trauma) app, version 1.1.1(125), showing the 4 main content areas (Home, Tools, Learn, and Progress) and an example question item screen from an in-app assessment.

Users can access the following four content areas: (1) the “Home” screen with inspirational quotes, user-selected bookmarks, and a programmed conversational feature for app guidance, (2) “Tools” for developing and practicing active coping skills, (3) “Learn” for psychoeducation topics and resources (eg, Veterans Crisis Line), and (4) “Progress” for tracking progress over time through in-app assessments and a goal-setting feature (Figure 1). The Tools section includes 54 coping tools (eg, “Managing Reactions” and “Finding My Values”). The Learn section includes 49 primary psychoeducation topics (eg, “Self-Blame and Anger” and “Relationships and Health”), and 29 subtopics embedded within the primary topics (eg, Types of Support). Features were designed based on Cognitive Behavioral Therapy (eg, Cognitive Processing Therapy) and Mindfulness-Based Therapy (eg, Acceptance and Commitment Therapy) principles and practices. Content is also informed by findings from MST research studies as cited within the “About” section of the app.

### Data Source

JavaScript Object Notation (JSON) formatted usage data were downloaded through a secure data management platform. This platform was developed by the VA National Center for PTSD for quality improvement purposes in accordance with VA’s Technical Reference Model [36]. All data collected were anonymous and, thus, did not include any identifying information about users, such as demographic information. The dataset comprised “event-level” data in which each row (or set of rows) of data represented one in-app event (ie, one app interaction, such as a button press or a swipe). The dataset included the following variables: (1) an anonymous computer-generated alphanumeric identification (ID) string assigned when the app was first downloaded, (2) the operating system (ie, Android or iOS), (3) coded event and metadata information describing the interaction (eg, which button was pressed on which screen), and (4) a timestamp indicating the date and time the event occurred.

ID numbers served as proxies for the identification of individual users. It is possible that multiple ID numbers were associated with a single user if they deleted and redownloaded the app, reset the app, or used the app on multiple devices. Given the inability to differentiate between actual users due to anonymity, and the likely rarity of such occurrences, unique ID numbers represented individual users for the purposes of this study.

### Procedure

Beyond MST usage data from March 11, 2021 through July 29, 2024, were downloaded on July 30, 2024. Data were cleaned, processed, and analyzed using R statistical software (v4.4.1; R Core Team). As part of data cleaning, errors in the raw event-level dataset were identified via descriptive statistics, graphical visualization, and simple univariate and bivariate analyses. Research and testing data were removed. Problematic data (eg, due to technical issues) were removed or corrected. Next, a data dictionary mapping user-app interaction data was constructed to guide summarization of the event-level data into user-level variables with one row per user. User-level variables captured total scores on in-app assessments, duration of app use, frequency of feature access, and frequency of feature use.

### Measures

#### In-App Assessments

Three measures were examined based on available usage data from the three assessments found in the Progress section of the app: (1) severity of PTSD symptoms, (2) severity of negative posttraumatic cognitions that are associated with PTSD symptoms, and (3) level of mental well-being.

#### Primary Measure: Severity of PTSD Symptoms

Severity of PTSD symptoms was measured using the in-app PTSD Checklist for *DSM-5* (*Diagnostic and Statistical Manual of Mental Disorders [Fifth Edition]*; PCL-5) [37]. This 20-item measure assesses how much an individual has been bothered by PTSD-related symptoms in the last 1 month. Items are rated on a 5-point Likert scale ranging from 0 (not at all) to 4 (extremely). Total scores range from 0 to 80 with higher scores indicating higher symptom severity. To help characterize the subsample of users who completed the in-app PCL-5, a cutoff score of ≥31 indicated a probable PTSD diagnosis [38]. However, the total PCL-5 score was included as a continuous variable in subsequent analyses. Internal consistency for this study was very good (Cronbach α=0.94).

A technical error found in the Android version of the app resulted in item 3 (“Suddenly feeling or acting as if the stressful experience were actually happening again (as if you were actually back there reliving it”) appearing twice, and item 4 (“Feeling very upset when something reminded you of the stressful experience”) missing from the in-app PCL-5. For Android users, responses to the first iteration of item 3 were retained. Due to item 4 being missing, total scores were prorated (for each user, their 19-item responses were averaged, then multiplied by 20). Finally, only scores from users’ first completion of the PCL-5 were included in analyses.

#### Primary Measure: Severity of Negative Posttraumatic Cognitions

Severity of NPCs was measured via the 15-item in-app Posttraumatic Maladaptive Beliefs Scale (PMBS) [11]. The PMBS is used to assess unhelpful and potentially harmful beliefs about current life circumstances that may develop following trauma exposure. Seven items represent potential maladaptive beliefs (eg, “I don’t feel safe anywhere anymore”) and 8 reverse-coded items represent potential healthy beliefs (eg, “I feel as though I can depend on other people”). Items are rated on a 7-point scale ranging from 1 (not true) to 7 (completely true). Total scores range from 15 to 105, with higher scores indicating more severe NPCs. Internal consistency in this study was good (Cronbach α=0.88). Scores from users’ first completion of the PMBS were included in analyses.

#### Secondary Measure: Mental Well-Being

A secondary measure of mental well-being was included as a comparator and to, more broadly, assess positive aspects of mental health in contrast to symptoms of mental illness. This was measured through the in-app 14-item Warwick-Edinburgh Mental Well-Being Scale (WEMWBS) [39]. Each item represents a positive feeling or behavior associated with general well-being (eg, “I’ve been feeling optimistic about the future,” or “I’ve been dealing with problems well”). Users reported the frequency at which each statement was experienced in the past 2 weeks on a scale from 1 (none of the time) to 5 (all of the time). Scores range from 14 to 70, with higher scores indicating higher levels of well-being. Internal consistency in this study was very good (Cronbach α=0.94). Scores from users’ first completion of the WEMWBS were included in analyses.

#### App Engagement

Measures of user engagement with the Beyond MST app were grouped into three categories: (1) duration of app use, (2) frequency of feature access, and (3) frequency of feature use. Duration of app use was assessed via 2 metrics for the number of distinct days and the number of minutes of app use within the observation period. For minutes of use, serial events greater than 10 minutes apart were recoded to indicate the end of a session and the start of the next session. This prevented overinflation when users navigated away from the app without shutting it down or ending the session. Frequency of feature access was assessed using 2 metrics for the frequency of tool access and the frequency of Learn (psychoeducation) topic access within the observation period. Finally, feature use was assessed using 1 metric for frequency of assessment completion (ie, the number of times the in-app PCL-5, PMBS, and WEMWBS were completed in total).

### Data Analytic Plan

#### Analytic Samples

The following three subsamples of users who completed the in-app symptom assessments were identified: (1) users who completed at least one PCL-5, (2) users who completed at least one PMBS, and (3) users who completed at least one WEMWBS. These subsamples were analyzed separately since users varied in terms of which assessments they completed. For each, we calculated the number of iOS and Android users, the number of users meeting criteria for subthreshold PTSD versus probable PTSD, and summary statistics related to assessment scores and app engagement. Due to the sampling method and properties of the outcome variables, the datasets did not have missing data—except for the previously noted missing value in PCL-5. Since PCL-5 scores for Android users were prorated, a Wilcoxon rank-sum test was also conducted to check for large differences in scores between Android and iOS users.

#### Variable Diagnostics

Predictor and outcome variable distributions were examined descriptively, statistically, and visually to inform model selection for predicting app engagement. Findings indicated significant deviations from normality, including positive skewness and overdispersion (ie, observed variance exceeding the expected variance under a Poisson model), violations of residual normality assumptions for linear regression models, as well as the presence of extreme outliers across all outcomes. For 4 outcomes (ie, distinct days of use, frequency of tool access, frequency of Learn topic access, and frequency of assessment completion), this was expected, as these are count variables. Most count variables have the majority of observations occurring at the lower end of the range, with fewer instances of higher counts, leading to a long right tail in the distribution.

Further examination of extreme outliers revealed that high counts reflected acceptable variations in user behavior (eg, “superusers,” repeated use over time) and were thus retained in the dataset. Winsorization, a statistical technique that reduces the potential effect of outliers by replacing values beyond a specified percentile with the nearest value within that percentile range, was applied to all outcome variables. A conservative approach was taken to address extreme outliers above the 99.7th to 99.9th percentile.

### Predicting App Engagement

Several types of models were constructed to correctly evaluate the impacts of symptom levels on app engagement metrics. Careful attention was given to the data type (ie, count and continuous) and distribution patterns of the outcome variables to inform the optimal model. Rather than include all 3 predictors in a single model, separate models were constructed for each of the 3 predictors on each of the 5 outcomes to maximize sample size and maintain power across the 3 subsamples. Additionally, a quadratic term was included in all models to assess for possible curvilinear relationships.

For 4 count variables (distinct days of use, frequency of tool access, frequency of Learn topic access, and frequency of assessment completion), Poisson and negative binomial models with log-links were evaluated. For distinct days of use and frequency of assessment completions, zero-truncated Poisson and negative binomial models were evaluated as a zero value was not possible. Model fit was compared based on the Akaike Information Criteria statistic, where lower index values indicate better fit [40]. Results from the zero-truncated and standard negative binomial regression models provided the best fit overall and for addressing overdispersion [40]. Thus, those modeling results were used to assess the impacts of predictors on outcome variables. Results are reported as unstandardized coefficients (B). To facilitate interpretation, coefficients were mathematically adjusted as relative percentage changes in expected app engagement using the transformation (*e*^B^ – 1) × 100.

For the continuous outcome, minutes of use, robust linear regression was used, which lessens the impact of outliers and nonnormality. Unstandardized regression coefficients (B) were interpreted as the expected change in minutes of use for each 1-point change in the predictor. All 3 predictors were mean-centered to reduce multicollinearity and simplify the interpretation of the regression coefficients.

**Figure 2:**
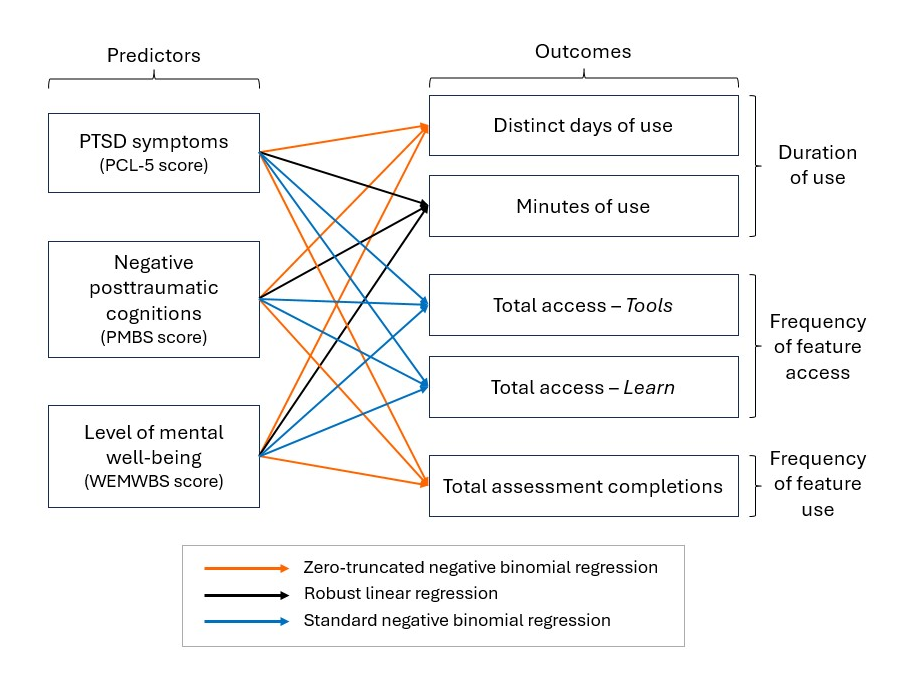
Conceptual models illustrating the predictors and outcome variables examined in a retrospective observational study of Beyond MST (military sexual trauma) app usage data. PCL-5: PTSD (posttraumatic stress disorder) Checklist for DSM-5 (Diagnostic and Statistical Manual of Mental Disorders [Fifth Edition]); PMBS: Posttraumatic Maladaptive Beliefs Scale; WEMWBS: Warwick-Edinburgh Mental Well-Being Scale.

Figure 2 illustrates the types of individual regression models selected. All analyses were performed using R statistical software (v4.4.1; R Core Team), with a significance level set at *P*<.05.

### Ethical Considerations

The Stanford University Institutional Review Board determined that this project is quality improvement and is not classified as human subjects research and was, therefore, excluded from oversight (protocol 75160).

## Results

### Sample Description

**Figure 3:**
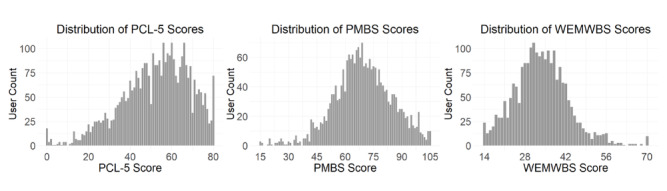
Distributions of PTSD (posttraumatic stress disorder) Checklist for DSM-5 (Diagnostic and Statistical Manual of Mental Disorders [Fifth Edition]) (PCL-5; n=3689), Posttraumatic Maladaptive Beliefs Scale (PMBS; n=2197), and Warwick-Edinburgh Mental Well-Being Scale (WEMWBS; n=2160) scores for subsamples of users who completed the Beyond MST (military sexual trauma) in-app assessments during the observation period.

Subsamples were obtained from a larger sample of users (N=27,517). Since the data were anonymous, sociodemographic information about users was not available. Among a subsample of users who completed at least one PCL-5 (3689/27,517, 13.4%), 1778 (48.2%) were Android users and 1911 (51.8%) were iOS users. Based on the PCL-5, a large majority of users (3293/3689, 89.3%) met criteria for probable PTSD (score of ≥31). In addition, the distribution of PCL-5 scores across users in this subsample was negatively skewed (Figure 3). To account for prorated responses in the PCL-5, a Wilcoxon rank-sum test was performed to assess potential differences in median PCL-5 scores between Android and iOS users. Results indicated a statistically significant difference in scores, W=1,622,530; *P*=.02, with median scores of 53.7 (IQR 22.1) for Android users and 55.0 (IQR 23.0) for iOS users. However, the effect size was small (*r*=0.04, indicating that 0.16% of variance in PCL-5 scores can be attributed to differences in Android vs iOS users), suggesting the observed difference (1.3 points) was statistically and clinically negligible. Consequently, data from different operating systems were combined for the main analyses. Descriptive statistics for all subsamples and engagement measures are shown in Table 1.

**Table 1:** Descriptive statistics for subsamples of Beyond MST (military sexual trauma) users based on completed in-app assessment.

Variable		Mean (SD)	95% CI	Median (IQR)	Range
**PCL-5^a^ subsample (n=3689)**					
	PCL-5 score	52.38 (16.25)	51.85-52.90	54 (22.89)	0-80
	Distinct days of use	6.43 (14.24)	5.97-6.89	3 (5)	1-416
	Minutes of use	46.58 (147.62)	41.81-51.34	22.15 (33.72)	0.45-5112.62
	Frequency of tool access	7.99 (20.77)	7.32-8.67	3 (7)	0-709
	Frequency of Learn topic access	5.14 (16.77)	4.60-5.68	1 (6)	0-637
	Frequency of assessment completion	2.80 (4.40)	2.66-2.94	2 (2)	1-151
**PMBS^b^ subsample (n=2197)**					
	PMBS score	69.49 (15.43)	68.84-70.13	69 (20)	15-105
	Distinct days of use	7.74 (17.61)	7.00-8.48	4 (6)	1-416
	Minutes of use	62.36 (188.87)	54.46-70.26	29.78 (42.85)	0.52-5112.62
	Frequency of tool access	11.27 (27.74)	10.11-12.43	5 (9)	0-709
	Frequency of Learn topic access	6.92 (23.34)	5.95-7.90	2 (7)	0-637
	Frequency of assessment completion	3.62 (4.47)	3.43-3.81	3 (2)	1-131
**WEMWBS^c^ subsample (n=2160)**					
	WEMWBS score	33.66 (9.39)	33.26-34.05	33 (11)	14-70
	Distinct days of use	7.96 (18.80)	7.17-8.76	4 (6)	1-416
	Minutes of use	61.74 (188.99)	53.76-69.71	29.21 (41.99)	0.75-5112.62
	Frequency of tool access	10.90 (27.02)	9.76-12.04	5 (9)	0-709
	Frequency of Learn topic access	6.55 (21.13)	5.66-7.45	1 (7)	0-637
	Frequency of assessment completion	3.84 (5.65)	3.60-4.07	3 (2)	1-151

^a^PCL-5: PTSD (posttraumatic stress disorder) Checklist for DSM-5 (Diagnostic and Statistical Manual of Mental Disorders [Fifth Edition]).

^b^PMBS: Posttraumatic Maladaptive Beliefs Scale.

^c^WEMWBS: Warwick-Edinburgh Mental Well-Being Scale.

Users who completed at least one PMBS (2197/27,517, 8%) included 1030 (46.9%) Android and 1167 (53.1%) iOS users. As with PCL-5 scores, the distribution of PMBS scores across users in this subsample was also negatively skewed (Figure 3).

Finally, the subsample of users who competed at least one WEMWBS (2160/27,517, 7.8%) included 1043 (48.3%) Android and 1117 (51.7%) iOS users. In contrast to the distributions for PCL-5 and PMBS scores, the distribution for WEMWBS scores was positively skewed (Figure 3).

### Relationship Between Trauma-Related Measures and App Engagement

#### Severity of PTSD Symptoms

**Figure 4:**
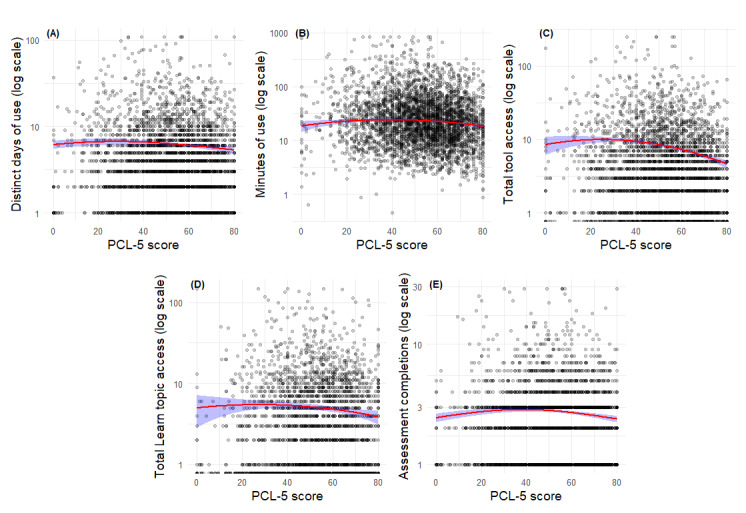
Predicted engagement outcomes by PTSD (posttraumatic stress disorder) Checklist for DSM-5 (Diagnostic and Statistical Manual of Mental Disorders [Fifth Edition]) (PCL-5) score for the subsample of Beyond MST (military sexual trauma) users who completed the in-app PCL-5 (n=3689).

Model results for PCL-5 scores are presented in Table 2 and graphs depicting the impacts of PCL-5 scores on outcomes are provided in Figure 4A-E. For distinct days of use, PCL-5 scores showed significant linear (B=−0.01, *z*=−2.7; *P*=.008), and quadratic (B=−0.0003, *z*=−3.2; *P*=.001) effects, such that days of use were lowest at very low symptom levels, highest at moderate PCL-5 levels, and declined again at high PCL-5 symptom levels. For 3 additional predictors of PCL-5 scores (ie, minutes of use, frequency of tool access, and frequency of assessment completion), similar inverted-U patterns of statistically significant negative linear (B<−3.1; *P*<.001) and quadratic (B<−0.004; *P*<.001) effects were found with the lowest and highest levels of PCL-5 scores predicting less engagement, access, and use, while moderate PCL-5 scores were related to increased levels of engagement, access, and use of the app. Finally, for frequency of Learn topic access, a 1-point increase in PCL-5 was linked to a 0.6% reduction in expected counts (B=−0.006, *z*=−2.8; *P*=.005), though the quadratic term was not significant (B=−0.0001, *z*=−1.3; *P*=.20), indicating a primarily linear relationship, where Learn topic access declined as PCL-5 scores increased. In summary, across 4 of the 5 metrics, moderate levels of PCL-5 scores were associated with the highest levels of engagement, access, and use, compared with lower and higher levels of symptoms.

**Table 2:** Results from models assessing relationships between PCL-5^a^ (n=3690), PMBS^b^ (n=2197), and WEMWBS^c^ (n=2160) scores and measures of app engagement for Beyond MST^d^ users who completed in-app assessments.

Predictor and outcomes			Linear term							Quadratic term				
			B^e^		Test statistic value^f^		*P* value		B			Test statistic value^f^		*P* value
**PCL-5**														
	Distinct days of use	–0.01		–2.7		.008		–0.0003			–3.2		.001	
	Minutes of use	–0.09		–4.5		<.001		–0.004			–4.0		<.001	
	Frequency of tool access	–0.01		–9.6		<.001		–0.0003			–4.1		<.001	
	Frequency of Learn topic access	–0.006		–2.8		.005		–0.0001			–1.3		.20	
	Frequency of assessment completion	–0.008		–3.1		.002		–0.0003			–4.9		<.001	
**PMBS**														
	Distinct days of use	–0.01		–2.2		.03		–0.0004			–2.7		.01	
	Minutes of use	–0.02		–0.7		.49		–0.002			–1.2		.22	
	Frequency of tool access	–0.008		–4.8		<.001		–0.0002			–2.3		.02	
	Frequency of Learn topic access	–0.003		–1.2		.23		–0.00009			–0.8		.40	
	Frequency of assessment completion	0.0005		0.31		.75		–0.00008			–1.9		.06	
**WEMWBS**														
	Distinct days of use	0.03		3.4		<.001		–0.0005			–1.7		.09	
	Minutes of use	0.11		1.9		.06		–0.004			–1.0		.30	
	Frequency of tool access	0.02		6.2		<.001		–0.0003			–1.5		.13	
	Frequency of Learn topic access	0.009		1.9		.06		0.0001			0.5		.62	
	Frequency of assessment completion	–0.003		–1.5		.13		–0.00009			–0.7		.49	

^a^PCL-5: PTSD (posttraumatic stress disorder) Checklist for DSM-5 (Diagnostic and Statistical Manual of Mental Disorders [Fifth Edition]).

^b^PMBS: Posttraumatic Maladaptive Beliefs Scale.

^c^WEMWBS: Warwick-Edinburgh Mental Well-Being Scale.

^d^MST: military sexual trauma.

^e^B: unstandardized coefficient.

^f^z-statistics are presented for zero-truncated negative binomial and standard negative binomial regression models. T-statistics are presented for robust linear regression models.

In Figure 4, red lines represent model-estimated values based on quadratic negative binomial, zero-truncated negative binomial, and robust linear regression models. Shaded blue regions indicate 95% CIs for predicted values. All plots display outcome variables on a log-transformed scale for interpretability. Panels A-E depict the results for PCL-5 score on each outcome: (A) distinct days of use, (B) minutes of use, (C) frequency of tool access, (D) frequency of Learn topic access, and (E) frequency of assessment completion.

#### Severity of Negative Posttraumatic Cognitions

**Figure 5:**
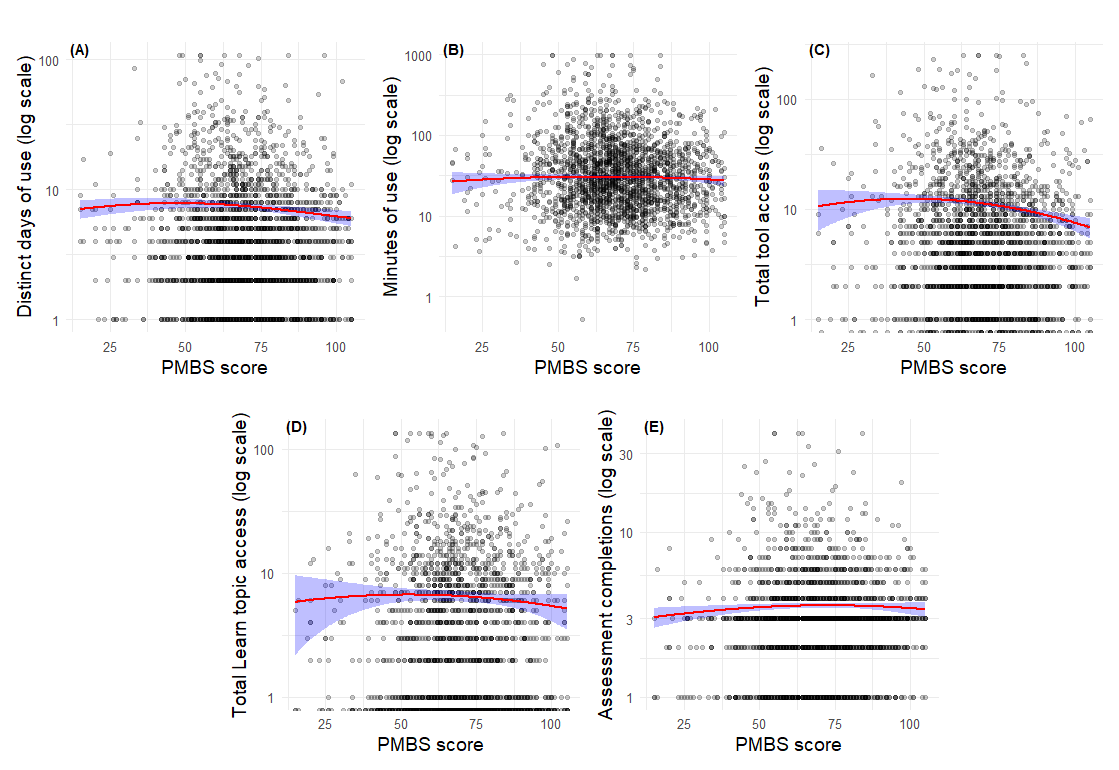
Predicted engagement outcomes by Posttraumatic Maladaptive Beliefs Scale (PMBS) score for the subsample of Beyond MST (military sexual trauma) users who completed the in-app PMBS (n=2197).

Table 2 and Figure 5A-E depict the results for the PMBS models. For distinct days of use, PMBS scores significant linear (B=−0.01, *z*=−2.2; *P*=.03) and quadratic (B=−0.0004, *z*=−2.7; *P*=.006) effects were identified. As with the PCL-5, this suggests that days of use were lowest at very low PMBS levels, highest at moderate PMBS levels, and declined again at high PMBS levels. A similar inverted-U pattern was observed for frequency of tool access, with statistically significant linear (B=−0.008, *z*=−4.8; *P*<.001) and quadratic effects (B=−0.0002, *z*=−2.3; *P*=.02). In contrast, statistically significant relationships were not observed between PMBS scores and the other 3 outcomes (ie, minutes of use, frequency of Learn topic access, and frequency of assessment completion; all *P*>.05). In summary, for days of use and tool access, moderate levels of PMBS scores were associated with the highest levels of engagement and access, compared with lower and higher levels of symptoms, while no relationships were found between PMBS scores and the other metrics.

In Figure 5, red lines represent model-estimated values based on quadratic negative binomial, zero-truncated negative binomial, and robust linear regression models. Shaded blue regions indicate 95% CIs for predicted values. All plots display outcome variables on a log-transformed scale for interpretability. Panels A-E depict the results for PMBS score on each outcome: (A) distinct days of use, (B) minutes of use, (C) frequency of tool access, and (D) frequency of Learn topic access, and (E) frequency of assessment completion.

#### Level of Mental Well-Being

**Figure 6:**
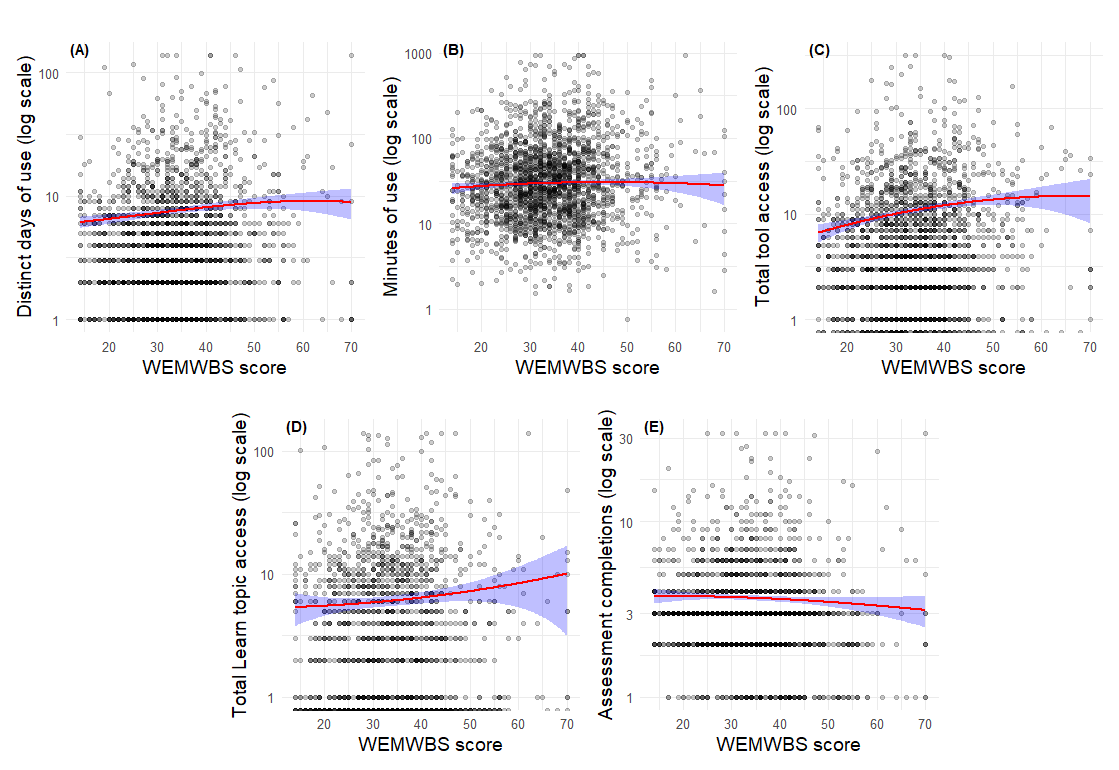
Predicted engagement outcomes by Warwick-Edinburgh Mental Well-Being Scale (WEMWBS) score for the subsample of Beyond MST (military sexual trauma) users who completed the in-app WEMWBS (n=1160).

Table 2 and Figure 6A-E depict the results for the WEMWBS models. In contrast to the PCL-5 and PMBS findings, higher WEMWBS scores, reflecting greater mental well-being, were associated with increased app engagement for 2 of the 5 outcomes. For distinct days of use, each 1-point increase in WEMWBS scores predicted a 3% increase in expected usage days (B=0.03, *z*=3.4; *P*<.001). A similar positive linear relationship was found for frequency of tool access, with a 2% increase in expected counts per 1-point increase in WEMWBS scores (B=0.02, *z*=6.2; *P*<.001). No significant effects were observed for minutes of use, frequency of Learn topic access, and frequency of assessment completion (*P*>.05). In summary, predicted usage days and frequency of tool access increased as WEMWBS scores increased, while no relationships were found for the other metrics.

In Figure 6, red lines represent model-estimated values based on quadratic negative binomial, zero-truncated negative binomial, and robust linear regression models. Shaded blue regions indicate 95% CIs for predicted values. All plots display outcome variables on a log-transformed scale for interpretability. Panels A-E depict the results for WEMWBS score on each outcome: (A) distinct days of use, (B) minutes of use, (C) frequency of tool access, (D) frequency of Learn topic access, and (E) frequency of assessment completion.

## Discussion

### Principal Findings and Comparisons With Prior Work

Mobile app engagement is a challenging construct to analyze, particularly when examining factors that influence human behavior in naturalistic settings. Researchers have made significant strides in understanding how aspects related to the apps themselves (eg, usability and acceptability) impact mental health app engagement. However, less is known about which user-level factors impact engagement. Emerging research highlights the potential influence of factors such as age, gender, and digital literacy [32]. However, few studies have evaluated how symptom severity might impact engagement with digital health technologies, especially mental health apps for trauma and PTSD. Studies examining other mental health conditions (eg, depression) suggest that symptom severity may play a crucial role [30-33]. This exploratory study furthers our understanding of how posttraumatic symptoms, negative cognitions, and levels of mental well-being may be associated with engagement with an app designed for those who experienced MST.

Based on usage data, more than 27,000 users engaged with Beyond MST from its launch on March 11, 2021, through July 29, 2024. A small subsample of users completed the in-app PCL-5 (3689/27,517, 13.4%), PMBS (2197/27,517, 7.8%), or WEMWBS (2160/27,517, 7.9%) at least once during the observation period. These assessment completion rates are consistent with data from studies on other publicly available mental health apps [33,41-43]. In addition, the subsamples were nearly split in terms of Android (46.9%-48.3%) versus iOS (51.5%-53.1%) users.

The results suggest that Beyond MST is reaching the targeted populations, including those with high levels of PTSD-related concerns, including negative posttraumatic cognitions and poorer mental well-being. Specifically, summary statistics for the subsamples revealed that scores for the PCL-5 and PMBS were positively skewed, indicating relatively severe PTSD symptoms and NPCs, whereas scores for the WEMWBS were negatively skewed, indicating relatively poor mental well-being overall. In addition, almost 90% (3293/3689) of users who completed the PCL-5 met criteria for probable PTSD. Taken together, it is likely that these subsamples of users are highly symptomatic, with substantial posttraumatic symptoms that may negatively impact their well-being and quality of life, similar to findings from studies of the PTSD Coach app [41,42].

Consistent with research on engagement with mental health apps, generally there was limited engagement with the Beyond MST app in terms of duration of app use as well as frequency of feature access and use. The median distinct days of use was just 3-4 days (IQR 5-6), and the median minutes of use was about 22-30 minutes (IQR 33.7-42.9) across samples. The median frequency of coping tool access and Learn (ie, psychoeducation) topic access was 3-5 (IQR 7-9) and 1-2 (IQR 6-7) times, respectively. Finally, the median frequency of assessment completion was 2-3 (IQR 2). Considering the tools are designed to be used multiple times to support coping skills practice and the assessments are meant to be completed repeatedly for progress tracking, it follows that median rates of tool access and assessment completion would be higher compared to psychoeducation access. Both the relatively low rates of engagement and the differences in tool versus psychoeducation access are consistent with findings from evaluations of other mental health apps with similar designs and content areas [33,41-43].

Results also uncovered curvilinear patterns related to trauma-related measures and app engagement. The nonlinear relationships were most clearly seen in relation to PTSD symptom severity and NPCs. For users with the most and least severe symptom levels (eg, PCL-5 score ≥65 or <25), engagement with Beyond MST was lower compared to users reporting moderate symptom levels (eg, scores of 30-40). These effects were consistent for 2 engagement outcomes, distinct days of use and frequency of tool access. This curvilinear pattern is consistent with the Yerkes-Dodson principle [44,45], describing the inverted-U shaped relationship between arousal and performance, with optimal levels of performance, or engagement in this case, at moderate levels of arousal and with decrements when arousal is too low or too high.

For minutes of app use and frequency of assessment completion, the relationship between symptom severity and app engagement was less consistent and clear. For minutes of app use, the results were mixed. Like with PTSD symptom severity and distinct days of use, there was a similar curvilinear relationship between PTSD symptom severity and minutes of use. However, no statistically significant relationship was found for NPC severity and minutes of use. It is unclear why consistent effects were not observed across similar duration of use metrics. One potential factor is the difference in the average minutes of app use between the subsamples (PCL-5 sample: mean 46.6, SD 147.6 minutes; PMBS sample: mean 62.4, SD 188.9 minutes) and increased variability. In contrast, consistent effects were found for distinct days of use where the subsamples had more similar means (PCL-5 sample: mean 6.4, SD 14.2 days; PMBS sample: mean 7.7, SD 17.6 days) with less variability.

These same patterns were observed for frequency of assessment completion where the highest and lowest levels of PTSD symptom severity predicted lower completion rates for all scales compared to those with moderate PTSD symptom levels, whereas NPC severity showed no significant linear or quadratic associations. Assessment completion reflects a complex, multistep behavioral sequence, requiring initiation, sustained attention, and follow-through. Therefore, it is possible that PTSD symptom severity may more strongly disrupt the cognitive and motivational processes needed to complete structured tasks, while NPCs alone may not exert the same level of interference. Other factors, such as the length of the scale (15 vs 20 items) and the trauma-related symptom content of the PCL-5 items may influence engagement.

The pattern of results for psychoeducation access was also inconsistent. There were no curvilinear relationships for either symptom severity measure. In addition, the results regarding linear effects were mixed. Increasing PTSD symptom severity predicted less psychoeducation access, but there was no relationship between NPC severity and psychoeducation access. Although the reasons for this discrepancy are unclear, it is possible that those experiencing particular PTSD symptoms at higher levels are seeking in-the-moment symptom relief versus wanting to learn about MST.

In contrast, greater mental well-being was associated with increased app engagement, generally following linear trends. These patterns were strongest for distinct days of use and tool access, while effects for psychoeducation access were more modest or inconsistent. Notably, those with the lowest levels of well-being accessed the tools less frequently compared to users with higher well-being. Taken together, this suggests that in this sample of primarily highly symptomatic users, individuals displaying better well-being–related factors (eg, positive emotions, life satisfaction, and functioning) may continue to find value in the app over time, using it for ongoing support; however, users with fewer internal resources may find slightly more difficulty sustaining engagement.

Overall, the novel findings of possible inverted-U patterns prompt a need for further assessment of curvilinear relationships in digital mental health engagement research. Potentially, users with lower posttraumatic symptom severity may not perceive a pressing need for support, while those with more severe symptoms may experience avoidance, emotional numbing, or cognitive overload that impairs their ability to engage meaningfully with digital tools. It also follows that emerging evidence of such relationships has been found for depression symptoms (eg, [33]) since depression often co-occurs with MST [5]. This also parallels psychotherapy research showing that both low perceived need and high avoidance can undermine treatment engagement and retention [46-48]. Therefore, it is possible that other symptoms not assessed are driving these relationships. Taken together, engagement is not simply a function of need, but also of readiness and psychological bandwidth.

However, it is important to interpret these findings in the context of clinical significance. According to the clinical guidelines for the PCL-5 [49], a 10-point change in the PCL-5 suggests a clinically significant change in PTSD symptoms. This can be used as a benchmark for understanding clinically meaningful differences in app engagement. For example, based on the model in this study, a 10-point increase on PCL-5 predicts approximately a 10% reduction in the expected number of distinct days of use, which translates to roughly half a day less engagement from a baseline median of 5 days. Similarly, for tools, psychoeducation, and assessments, a 10-point increase corresponds to modest decreases in the expected frequency of access or use. While these effects are statistically significant, the absolute changes in engagement are small, suggesting that even clinically meaningful differences in PTSD symptoms may only slightly reduce platform use. These small effect sizes highlight the importance of interpreting behavioral outcomes in real-world contexts where even brief or infrequent engagement may still yield therapeutic benefit. It is also possible that symptom levels indirectly affect engagement through more direct predictors such as motivation level [50]. This is especially pertinent for types of engagement that require sustained attention and repeated behavior, such as in-app symptom tracking over time or tool use for ongoing skills practice. Finally, since the subsamples displayed high levels of symptoms overall, it is important to interpret these findings in the context of highly symptomatic individuals. For example, compared to users with high levels of symptoms who may still benefit from self-guided use, perhaps those with the highest levels of symptoms might display improved engagement when provided additional support (eg, human coaching) when using apps such as Beyond MST.

Given the curvilinear relationships found, future work may also benefit from using analytic methods capable of detecting more complex or heterogeneous patterns of engagement. The current models tested only linear and quadratic associations assuming a homogeneous population. However, the overdispersion and presence of outliers suggest that the sample may include multiple populations, such as “typical users” and “super users,” who engage with the app differently. Future studies could use alternate methods, such as mixture modeling approaches to determine whether subgroups of users follow distinct engagement patterns or whether more nuanced relationships between symptom levels and app engagement exist.

In sum, PTSD symptom severity and, to a somewhat lesser degree, negative posttraumatic cognitions, may be important factors to consider when addressing user engagement with an app for MST. This has important implications for app developers and mental health professionals. Specifically, personalized in-app treatment plans or stepped care pathways based on baseline symptom level, such as increased human support for those with more severe symptoms, may improve app engagement. Providers using apps in care should also consider symptom severity when making recommendations (eg, psychoeducation vs tool use). Additionally, the insights generated from these analyses suggest how symptom severity can be used to understand digital interventions along the continuum of care. This is especially crucial for apps such as Beyond MST that can be systematically integrated with care in large health care systems serving diverse populations with a range of mental health symptoms and trauma types [51]. Although these findings may suggest potential clinical implications for the subsamples examined, limitations of the current study should be considered when interpreting these results more broadly.

### Limitations

Several study limitations are inherent to the nature of the data collected as well as the subsamples and methods used. First, due to the data being anonymous, user characteristics were not accessible, making it impossible to control for potential confounding factors such as sociodemographics. This may have been problematic because some demographic factors (eg, female gender [4] and young age [52]) are disproportionately associated with risk of MST victimization. More generally, studies have found that women are also more likely to adopt and engage with digital mental health interventions compared to men [32]. Therefore, it is possible that the subsamples examined in this study were not demographically diverse, limiting the generalizability of these results to the broader population. Clinical trials should be conducted to explore the relationship between posttraumatic symptom severity and app engagement while controlling for potential confounding factors.

Second, this study focused on a subset of users who engaged with the in-app assessments, which may limit generalizability to the complete user sample. This potentially biases the results, as these subsamples of users may be more engaged when compared to the overall user base. Nevertheless, there remains value in the evaluation. First, the data provide insights into the real-world use patterns and relationships among large user samples that engaged with a free and publicly available app. For-profit companies often do not release such detailed information around mobile app engagement. These results can inform hypothesis generation for future studies and potential methods for increasing engagement. Regardless, further user evaluation and controlled studies are needed to identify possible differences in engagement between the broader sample of users and the subsets examined in this study.

Third, the in-app assessments and measures of app engagement used in this study may not fully capture the scope of posttraumatic symptom severity and its effects on app engagement. Several different symptoms are associated with trauma and PTSD, so the use of PCL-5 scores does not inform how individual posttraumatic symptoms might predict app engagement. For example, avoidant behavior, a hallmark symptom of PTSD, might negatively impact app engagement when compared to other symptoms [5]. Also, this study focused on objective metrics of app engagement. Future user experience studies with Beyond MST should explore subjective measures of app engagement such as self-reported intentions to engage. This would provide a more comprehensive picture of how users intended to engage, how they actually engaged, and possible barriers to engagement.

Relatedly, there are numerous app engagement measures that were not explored due to the constraints of the app’s design and availability of data. For example, apart from feature access and assessment feature use, other types of feature use could not be assessed due to limitations in the backend of Beyond MST. Importantly, researchers have urged the field to move beyond just measuring “clicks and downloads” when examining app engagement [53]. Engagement is a multidimensional construct that not only includes behavioral aspects (eg, duration of use and frequency of use), but also subjective experiential aspects (eg, focused attention, emotional involvement, and usability) [53] which are often best understood through qualitative and mixed methods approaches. The extent to which users meaningfully apply skills learned or practiced via the app in day-to-day life should also be explored in future work. For mental health apps, this means defining what constitutes clinically meaningful app engagement. For example, the frequency of accessing the Deep Breathing tool in Beyond MST does not fully capture the experiences of users who adopted the skill with minimal app use but continued to apply that skill in daily life. In other words, clinically meaningful app engagement may vary from person to person. Longitudinal studies are also needed to understand how symptom severity may impact engagement over time. Overall, further research is needed that captures the breadth of these complex constructs, providing a more nuanced understanding of how posttraumatic symptoms might impact mental health app engagement.

### Conclusions

The Beyond MST app has the potential to support a diverse range of service members and veterans who experienced military sexual trauma, whether they are using the app for self-care purposes, or are using it in conjunction with professional care. Even highly symptomatic users were found to engage with Beyond MST. However, some small effects on user engagement based on posttraumatic symptom severity levels were found which may limit the potential effectiveness of Beyond MST in helping some users cope with trauma, find hope, and enhance their well-being, particularly users with the highest levels of symptoms. Additional research on possible user-level predictors of app engagement, and how specific factors might promote or impede engagement, is needed. Findings from this study suggest statistically significant curvilinear and linear relationships between posttraumatic symptom severity and app engagement, but the clinical implications of these results remain unclear without additional study. Careful examination of the potential predictive power of individual posttraumatic symptoms on a wider range of app engagement measures is needed. Nevertheless, this study may serve as a model for future research in this area and highlights the importance of understanding symptom severity as it relates to user engagement to optimize digital solutions for mental health conditions. Continued exploration in this area will be crucial for developing more effective mental health apps for people who have experienced MST and other types of traumatic stress.

## Data Availability

The participants in this quality improvement project did not provide written consent for their data to be shared publicly. Therefore, due to the sensitive nature of the research, supporting data are not available.

## Abbreviations

DSM-5: Diagnostic and Statistical Manual of Mental Disorders, Fifth Edition
ID: identification
JSON: JavaScript Object Notation
MST: military sexual trauma
NPC: negative posttraumatic cognition
PCL-5: PTSD (posttraumatic stress disorder) Checklist for DSM-5 (Diagnostic and Statistical Manual of Mental Disorders [Fifth Edition])
PMBS: Posttraumatic Maladaptive Beliefs Scale
PTSD: posttraumatic stress disorder
VA: US Department of Veterans Affairs
WEMWBS: Warwick-Edinburgh Mental Well-Being Scale
